# Immune Cell Modulation of Patient-Matched Organoid Drug Response in Precision Cancer Medicine Platform

**DOI:** 10.3390/cells15030259

**Published:** 2026-01-29

**Authors:** Silje Kjølle, Mario Presti, Jéssica de Pina Roque, Lina Hua Bisgaard, Darío Beceiro Ramos, Kamilla Westarp Zornhagen, Christina Westmose Yde, Ane Yde Schmidt, Perrine Verdys, Martin Højgaard, Ulrik Lassen, Inge Marie Svane, Kristoffer Staal Rohrberg, Marco Donia, Janine T. Erler

**Affiliations:** 1 Biotech Research and Innovation Centre (BRIC), University of Copenhagen (UCPH), 2200 Copenhagen, Denmark; silje.kjolle@bric.ku.dk (S.K.);; 2 National Center for Cancer Immune Therapy, Department of Oncology, Copenhagen University Hospital, 2730 Herlev, Denmark; mario.presti.01@regionh.dk (M.P.);; 3 The Phase I Unit, Department of Oncology, Rigshospitalet, 2100 Copenhagen, Denmark; 4 Center for Genomic Medicine, Rigshospitalet, 2100 Copenhagen, Denmark

**Keywords:** precision medicine, metastasis, immunotherapy, 3D co-culture platform

## Abstract

Cancer is one of the leading causes of death worldwide, and the majority of cancer-related deaths are caused by cancer that has spread to other organs. Precision cancer medicine (PCM) holds potential to improve outcomes and relies on molecularly matched therapies based on cancer cell specific molecular alterations. The tumor immune microenvironment plays an important role beyond response to therapy; however, this is generally not considered in current PCM platforms. We established patient-matched organoids and immune cell cultures for drug testing in mono- and co-culture treatment setups using three distinct treatment strategies (pretreatment, co-culture treatment, and T-cell bispecific antibody testing). Response to treatment and impact of immune cells were evaluated by tumor cell viability assays and flow cytometry analysis. Phenotypic analysis showed high heterogeneity of tumor-infiltrating lymphocytes (TILs) across the patients and low immune cell portions of organoids, emphasizing the need for a patient-matched co-culture PCM approach. Our in-depth study of three patients revealed an effect of the patients’ immune cells on drug response and T-cell bispecific antibody treatment in vitro. Here, we illustrate a state-of-the-art co-culture PCM pipeline for patient-matched organoids and immune cells replicating patient response to treatment at the time of biopsy.

## 1. Introduction

The large heterogeneity among cancer patients as well as within tumors, causes the ‘one-size fits all’ approach to therapy to be largely ineffective, with an increasing need for precision cancer medicine (PCM) pipelines. In particular, late-stage and advanced cancers often show poor response to treatment [1,2]. Genomic profiling has been shown to be useful for suggesting treatment targets for patients with advanced cancers and exhausted treatment options [3,4,5]. However, this approach only suggests therapies for 20% of patients with about 50% efficacy [6,7].

The tumor context is increasingly recognized as an important determinant of therapeutic treatment response [8,9]. The immune cells of the tumor microenvironment (TME) have been found to play an important role not only in response to immunotherapies, but also in affecting non-immune agents [10,11], through cytokine secretion, immune checkpoint interactions and induction of immunogenic cell death [12,13]. Ignoring these interactions limits predictive accuracy and may lead to suboptimal treatment selection. Recent studies have demonstrated that co-culture systems incorporating immune cells better recapitulate patient-specific responses and resistance mechanisms [14,15,16,17,18,19].

Current PCM platforms are generally limited to drug testing on patient-derived organoids (PDOs) in vitro, which excludes other cells of the TME, or are dependent on the establishment of patient-derived xenograft (PDX) mouse models, which often take longer to establish than the patient’s survival. There is growing interest in co-culture systems due to their benefit in recapitulating therapy response [14,15]. Indeed, PDO and TIL co-culture have previously been used to assess TIL reactivity and tumor killing [20,21]; however, there are no standardized methods for such setups.

Here, we process tumor biopsies for establishing patient-matched organoid and immune cell co-cultures. We develop an in vitro PCM drug testing platform to suggest treatment for patients, and further build on this setup for investigations of the tumor immune microenvironment and its role in drug response. As the relevance of immunotherapy continues to rise, we extended the applicability of our platform by adapting it for the testing of immune agents. Our study illustrates the need to include TME components in PCM pipelines and platforms to replicate patient response to treatment, with the potential for implementation in the treatment of patients with less advanced cancers.

Our platform addresses these limitations by integrating patient-matched PDOs with autologous TILs in a versatile co-culture system. This setup enables the functional testing of chemotherapy, targeted therapy and immunotherapies (including T-cell bispecific antibodies) within a patient-specific context. By replicating the immune–tumor interface, the platform provides immediate translational potential for guiding personalized combination therapies and informing rapid clinical decisions for patients with advanced disease.

## 2. Materials and Methods

### 2.1. Study Design and Objectives

The PreCanIO is a prospective, single-center, single-arm open-label study enrolling patients at the Phase I Unit at Rigshospitalet, University of Copenhagen (Copenhagen, Denmark), including patients in the CoPPO trial (NCT02290522) [3,22]. The study was conducted according to the Declaration of Helsinki. Regulatory approvals from the Regional Ethics Committee and the Danish Data Protection Agency were obtained (Danish Ethical Committee, file number: 1300530). All patients signed informed written consent.

### 2.2. Patient Inclusion and Biopsy Procedure

Patients were enrolled from March 2023 to May 2025. Patients with advanced solid malignancies referred to the Phase I Unit at Rigshospitalet were offered enrolment. Eligibility criteria were exhausted treatment options, life expectancy of ≥3 months, normal organ function, measurable disease (RECIST1.1), Eastern Cooperative Oncology Group (ECOG) performance status of 0 or 1, age ≥ 18 years, and lesions safely accessible for biopsy (Table 1).

Fresh tumor samples, primarily from metastatic sites, were obtained under local anesthesia. Biopsies were either core-needle biopsies (18-gauge needle) or surgical resection samples.

For genomic profiling, tumor tissue preserved in RNAlater was obtained for WGS and RNA sequencing. Two 18G core needle biopsies (or similar amount of surgically resected tissue) for organoid and immune cell cultures were obtained from the same metastatic tumor lesion and collected into DMEM/F-12 (Thermo Fisher Scientific, Waltham, MA, USA) supplemented with penicillin–streptomycin (PS; 10,000 U/mL; Life Technologies, Carlsband, CA, USA), amphotericin B (Thermo Fisher Scientific, Waltham, MA, USA), and gentamicin (Thermo Fisher Scientific), and transported on ice for immediate biopsy processing for organoid and immune cell culture. Needle biopsies were cut into 2–3 mm fragments and alternately divided into cancer organoid or immune cell cultures.

### 2.3. Genomic Profiling

Genomic analyses were conducted for each patient as previously described [22]. Briefly, DNA and RNA were extracted from tumor biopsies using AllPrep DNA/RNA/Protein Mini Kit (QIAGEN, Redwood City, CA, USA). In addition, germline DNA was extracted from whole blood samples using the DSP DNA kit on a QiaSymphony workstation (QIAGEN). Library preparation for WGS was performed using Illumina PCR-free prep (Illumina). For RNAseq, library preparation was performed using the Illumina Stranded Total RNA kit. Sequencing was undertaken on either NovaSeq6000 or NovaSeqXPlus (Illumina, San Diego, CA, USA).

Somatic mutations were called by a Strelka2 variant caller from the tumor-normal analysis of WGS data. Filtration of somatic mutations was undertaken in VarSeq v2.6.2 (Golden Helix, Bozeman, MT, USA) and classification was based on criteria from Horak et al. 2022 [23]. Tumor mutational burden (TMB) was calculated as the number of non-synonymous somatic mutations per megabase (mut/Mb). Copy number variations (CNVs) were assessed from the genomic profiles. Homologous recombination deficiency (HRD) status was determined as previously described [24]. Expression levels of selected genes were calculated from RNAseq data, normalized to average expression in a large cohort of cancer patients from the CoPPO study. Germline variants were called using Haplotype caller from germline WGS data and variant classification was undertaken according to the ACMG/AMP guidelines.

The results from the genomic profiles were used for guiding the drug testing on organoids.

### 2.4. Biopsy Processing for Cancer Organoid Culture

For organoid cultures, tissue fragments were further minced and processed as previously described [25,26]. Processed biopsies were maintained for up to 6 months for the growth and development of organoids. Patient-derived organoid (PDO) success was defined as an organoid-like growth pattern, cells tolerating passaging for expansion of material, and sufficient growth rate to obtain material for drug testing.

For the passaging of organoids, the domes of basement membrane extract (BME) were collected in cold phosphate buffered saline (PBS; Sigma-Aldrich, St. Louis, MO, USA) to dissolve the BME and were pelleted by centrifugation (1400 RPM, 5 min; Multifuge© 3S, Kendro Laboratory Products, Asheville, NC, USA). The organoids were manually disrupted using a pipette before adding BME and plating new domes. All experiments were performed using organoids at maximum passage 10. Organoids were negative for Mycoplasma during regular testing.

### 2.5. Biopsy Processing for Immune Cell Culture

Biopsy processing for immune cell cultures and generation of young TILs (yTILs) was performed as previously described [27,28]. Briefly, the tumor fragments were plated individually in 24-well plates (VWR) in 2 mL complete medium consisting of RPMI-1640 (Life technologies, Carlsband, CA, USA) supplemented with 10% heat-inactivated human AB serum (HS; Sigma-Aldrich), 100 U/mL PS, 1.25 µg/mL Fungizone (Bristol-Myers Squibb, New York, NY, USA) and 6000 IU/mL recombinant human IL-2 Proleukin^®^ (Novartis, Basel, CH) and maintained for 4–6 weeks. If yTILs reached confluency, the yTILs were resuspended and divided between two new wells with fresh complete medium.

At week 6 at the latest, all yTIL material was collected and passed through a 70 µm Corning^®^ Cell Strainer (Corning, New York, NY, USA) to remove tumor fragments. Cells were counted using a hemacytometer and 1:1 mixing ratio with 0.2% Erythrosin B (Sigma-Aldrich, St. Louis, MO, USA) to determine cell viability. If the yTIL yield was higher than 1,000,000 cells, cells were cryopreserved in HS supplemented with 10% DMSO (WAK-chemie, Steinbach, Germany).

yTIL culture success was defined as achieving yTIL outgrowth in at least one well of a 24-well plate and cryopreservation of at least one cryotube of yTILs following initial culture.

### 2.6. Immune Cell Expansion

yTILs were expanded using the rapid expansion protocol (REP) as previously described [27,29]. For the expansion of yTILs from frozen vials, yTILs were thawed 2 days before REP initiation. Frozen vials were thawed in a 37 °C water bath and transferred to pre-warmed RPMI 1640 media (Life Technologies, Carlsband, CA, USA) containing 0.025 mg/mL Pulmozyme^®^ (Roche, Basel, CH) and 2.5 mM MgCl_2_. The cells were centrifuged (300× *g*, 5 min), washed with PBS, and centrifugation was repeated. Cells were plated in 24-well plates at 3–4 × 10^6^ cells/well with 2 mL of RPMI 1640 supplemented with PS, 10% HS, 6000 IU/mL IL-2 and 1.25 µg/mL Fungizone^®^ (Bristol-Myers Squibb, New York, NY, USA). For expansion directly from freshly isolated yTILs, the REP was initiated on the same day as cell harvest.

### 2.7. Isolation of Feeder Cells

Feeder cells were prepared from buffy coats obtained from Odense University Hospital and used to support T cell expansion. Peripheral blood mononuclear cells (PBMCs) were isolated using a density gradient centrifugation using Leucosep^TM^ tubes (VWR, Radnor, PA, USA) pre-filled with Lymphoprep^TM^ (STEMCELL, Vancouver, BC, Canada) and centrifuged (1000× *g*, acc. 9, dec. 3 for 1 min). Blood was diluted 1:4 in PBS, poured into the Leucosep^TM^ tube and centrifuged (1000× *g*, acc. 9, dec. 3, for 10 min). The supernatant containing the PBMCs layer was collected and washed with PBS at centrifugation (300× *g*, acc. 9, dec. 9, for 5 min), pooled by the donor, and subsequently combined into a single feeder cell batch. Cells were counted using the Sysmex XP-300^TM^ (Sysmex, Kobe, Japen), and PBMCs were resuspended in heat-inactivated HS with 10% DMSO (WAK-chemie) and cryopreserved at a concentration of 1 × 10^8^ or 3 × 10^8^ cells/mL per cryovial. Cryovials were frozen at −80 °C overnight in a CoolCell^TM^ container (Buch & Holm A/S, Herlev, Denmark), then transferred to a −140 °C freezer for long-term storage.

### 2.8. Rapid Expansion Protocol

On day 0 of the REP, 0.1 × 10^6^ yTILs were co-cultured with 20 × 10^6^ irradiated (40 Gy) allogeneic feeder cells in T25 cell culture flasks (VWR) with a 1:1 mix of complete medium and rapid expansion medium (RM) consisting of AIM-VTM (Life Technologies) supplemented with 6000 IU/mL human IL-2 Proleukin^®^ (Novartis), 1.25 µg/mL Fungizone^®^ (Bristol-Myers Squibb), supplemented with 10% HS. Additionally, anti-CD3 antibody (30 ng/mL, OKT-3; Miltenyi, Bergisch Gladbach, DE was added to promote T cell activation. As a control, a T25 flask with only feeder cells was included. The T25 flasks were incubated in an upright position. On day 5, half of the media was replaced with fresh RM. From day 6 to day 14, cultures were inspected daily and flasks were gently shaken. If cell confluence was observed, fresh RM without HS was added, and cells were expanded into larger culture flasks (T80 or T175; VWR) if needed. On day 14, cells were collected, pooled, and counted. Depending on yield, cells were cryopreserved in HS with 10% DMSO as described above for yTILs, and/or used for functional assays. TILs that underwent 12–14 days of expansion are referred to as REP TILs. REP TIL culture success was defined as generation of at least 10 × 10^6^ cells.

### 2.9. Characterization of Cells in PDOs by Flow Cytometry

Organoids were harvested as described above. To dissociate organoids from BME, the cell pellet was treated with 200 μL of pre-warmed Dispase^®^ II (2 mg/mL in PBS, Sigma-Aldrich), and incubated for 15 min at 37 °C. Dispase activity was then inhibited by the addition of 10% of EDTA (100 μL of 0.5 M EDTA for every 1 mL Dispase). PBS was added in excess prior to centrifugation (300× *g*, 5 min). Then, 1 mL of TrypLE (Fischer Scientific) was added to the cell pellet to dissociate the PDOs into a single-cell suspension. PBS was added, centrifugation was repeated, and the cells were plated by dividing the cell pellet into two wells in a 96-well V-bottom plate (Sigma-Aldrich).

After plating, cells were washed with wash buffer (0.005% bovine serum albumin (BSA), Sigma-Aldrich; 0.4% EDTA, Thermo Fisher Scientific, Waltham, MA, USA), and FBS-PBS supplemented with 0.005% NaN_3_ (Pharmacy, Herlev Hospital, Cat. No. 800979). The supernatant was discarded, and 20 μL of the NIR/FC-blocker premix was added to each well. The NIR/FC-blocker premix consisted of NIR dye (Invitrogen, Carlsband, CA, USA) dissolved in DMSO and Fc-blocking solution prepared in FACS buffer (incl. NaN_3_) with human IgG (100 mg/mL Kiovig immunoglobulin; Takeda, Tokyo, JP) at a final concentration of 1 mg/mL IgG. Cells were incubated for 10 min at 4 °C in the dark. Then, 30 μL of the antibody mixture (Panel 3 or 4; Supplementary Table S1) was added and incubated for 20 min at 4 °C in the dark. Cells were washed three times with wash buffer prior to resuspension in 100 μL of wash buffer before running the samples on the NovoCyte Quanteon Flow Cytometer to acquire the fluorescence intensity from each stained marker pr. event.

### 2.10. T Cell Characterization by Flow Cytometry

Both yTILs and REP TILs, from fresh or thawed material, were used for phenotyping analysis. From frozen material, TILs were thawed as described above. The same batch of irradiated feeder cells was used for all REP-generated T cells in this study.

TILs were harvested by collecting all the cells and centrifuged (300× *g*, 5 min). Prior to antibody staining, 500,000 viable cells were transferred into a 96-well V-bottom plate (Corning^®^) at a density of 250,000 cells/well. Subsequently, 25,000 cells from each well were transferred to separate wells for unstained controls. Moreover, to detect staining variation across experiments, PBMCs from the same batch were stained with Panels 1 and 2 (Supplementary Table S1) at each staining. TILs were washed and stained with antibody mixture (Panel 1, Panel 2, or unstained control panels; Supplementary Table S1) as described above, prior to acquisition on the NovoCyte Quanteon Flow Cytometer (Agilent, Santa Clara, CA, USA).

FlowJo v10.10.0 was used for data preprocessing, and data were imported into R. For each patient, a subsample of 10,000 events was selected and clustered using FlowSOM in the Spectre package (v1.2.0) and UMAP for visualization. Clusters were annotated based on marker expression, with CCR7 and CD45RA used to define T cell memory subsets. Additional markers included HLA-DR, CD57, and PD-1 (Panel 1) to indicate T cell activation, senescence, or exhaustion, respectively. In Panel 2, cells positive for more than one of the exhaustion markers (LAG-3, BTLA, TIGIT, TIM-3, or PD-1) were annotated as exhausted. Raw data are provided in the Supplementary File S1.

### 2.11. Patient Selection for PreCanIO Pipeline Description

**Figure 1:**
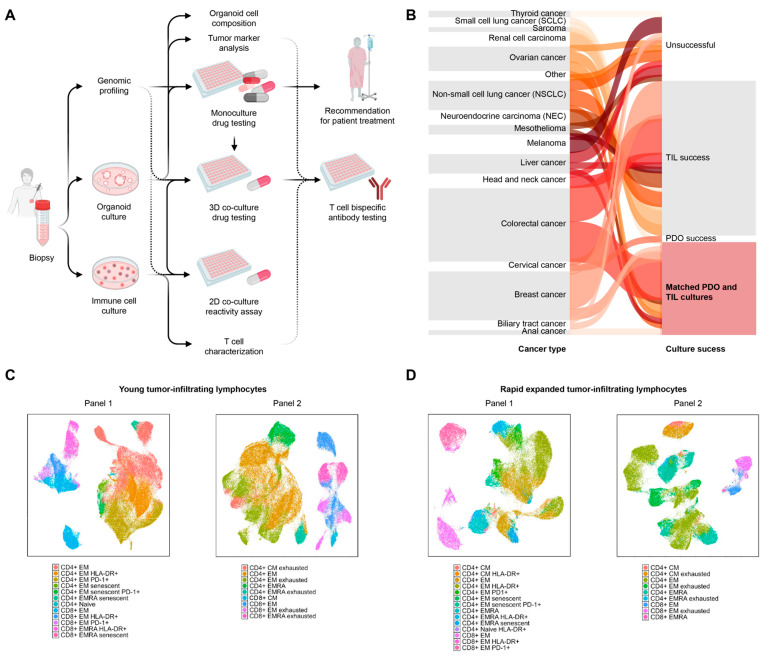
PreCanIO overview. PreCanIO pipeline (**A**), showing analysis flow from biopsy to clinical treatment recommendation, 2D reactivity and 3D co-culture drug response assays, including T-cell bispecific antibody testing. Parallel categories plot for primary cancer type of PreCanIO patients (*n* = 66) and technical success of biopsy processing into organoid and immune cell cultures (patient-matched/PDO/TIL culture success) (**B**). UMAP analysis demonstrates phenotypic heterogeneity across both yTILs (*n* = 14) (**C**) and REP TILs (*n* = 10) (**D**). Cells were clustered using FlowSom and annotated based on marker expression from panel 1 (left) and panel 2 (right). Parallel categories plot was created using pyalluvial in Python (v3.12). UMAPs created using Spectre in R (v10.10.0). CM: central memory. EM: effector memory. EMRA: terminally differentiated effector memory. PDO: patient-derived organoid. REP: rapid expansion protocol. TIL: tumor-infiltrating lymphocyte. yTIL: young TIL.

Three patients were used for this in-depth study, selected from the patients where we successfully obtained both organoid and immune cell cultures. One patient was alive at the time organoids had successfully developed and was subjected to the complete PreCanIO pipeline (Figure 1A), and two additional patients (deceased) were selected for methodological supportive purposes (Table 2). Relevant drugs were selected based on the genomic profile from each patient (Table 3).

### 2.12. Drug Screening for Treatment Recommendation

If the patient was alive when processed biopsies were successfully growing organoids, a drug screening was performed to suggest treatment for the patient. Organoids were plated 3 days prior to the addition of drugs, and treated for 3 days. For plating, organoids were harvested in cold PBS and pelleted by centrifugation (1400 RPM, 5 min; Multifuge© 3S, Kendro Laboratory Products). Organoids were manually disrupted using a pipette prior to manual counting and plated at a concentration of 10,000 cells for each 10 µL dome of BME in a 96-well plate setup with triplicates for each condition. For each well containing domes, 100 µL basal organoid media without growth factors was added, and 200 µL PBS was added to the outer wells.

Drugs were selected specifically based on the patient’s genomic profile. If the genomic profile did not provide an indicator for any drug, up to five drugs were selected from a panel of kinase inhibitors (Supplementary Table S2). The screening setup was standardized, and the same concentrations were used for all drugs (10 µM, 1 µM, 100 nM). Drug concentrations were selected based on clinically relevant plasma levels reported in pharmacokinetic studies and validated in prior in vitro dose–response experiments, where available, covering the majority of kinase inhibitor ranges (Supplementary Table S2). This range captures therapeutic exposures while enabling detection of dose-dependent effects. The drug stock solution (10 mM in DMSO) was diluted in basal organoid medium without growth factors.

Viability was measured using PrestoBlue^TM^ HS Cell Viability Reagent (Thermo Fisher Scientific). To each well containing 100 µL organoid media, 11 µL PrestoBlue reagent was added and incubated for 30 min, 37 °C, protected from light. Readings were obtained for 560 nm and 590 nm using SpectraMax^®^ i3 (Molecular Devices, San Jose, CA, USA) and SoftMaxPro (v7.1). Viability was measured on day 0 prior to the addition of drugs, and on day 3. On day 0, media containing PrestoBlue reagent was removed, and wells were washed with 100 µL warm PBS twice and incubated with basal organoid medium for 20 min, 37 °C, before adding fresh media with the drugs.

Drug response was compared with a negative control (0.1% DMSO; Sigma-Aldrich), and a positive control (20% DMSO). Indications of response were reported to the clinic for possible consideration in treatment selection for the patient.

### 2.13. Drug Screening and Evaluation of Immune Cell Tumor Reactivity and Effect on Treatment Response

REP TILs were thawed as described above and subsequently counted and divided into rested or activated conditions. Rested REP TILs were plated in a 24-well plate with RPMI supplemented with PS and 10% HS.

### 2.14. T Cell Activation

Dynabeads^®^ Human T-Activator CD3/CD28 (Thermo Fisher Scientific) were used to activate the TILs. The Dynabeads^®^ were thoroughly resuspended by vortexing for at least 30 s. The required volume was mixed with an equal volume of cold MACS buffer (PBS with 0.1% BSA and 2 mM EDTA) for washing. The suspension was mixed by vortexing for 5 s, and beads were isolated by placing the tube on a magnetic separator for 1 min and resuspended in RPMI supplemented with PS and 10% HS at a volume equal to the original volume taken from the stock.

The prepared CD3/CD28 beads were added to the TIL suspension for activation at a 1:1 bead-to-cell ratio and seeded into tissue culture plates. Rested and activated REP TILs were incubated for 1 day at 37 °C with 5% CO_2_.

### 2.15. Two-Dimensional Immune Cell Reactivity Assay

Anti-tumor reactivity of TILs was assessed by co-culturing REP TILs with autologous tumor cells derived from PDOs, followed by intracellular and surface staining for T cell activation markers (CD107a, TNF, IFN-γ, CD137; Panel 5, Supplementary Table S1) to identify tumor-reactive T cells [29,30].

Three days prior to the initiation of co-culture experiments, organoids were harvested as described above. Organoids were washed with cold PBS and pelleted by centrifugation (300× *g*, 5 min). Remaining BME was dissolved by adding dispase (2 mg/mL) as described for characterization of cells in PDOs. Dispase was inhibited by dilution with RPMI 1640 supplemented with PS and 10% HS, followed by centrifugation at 300× *g* for 5 min. Organoids were resuspended in RPMI 1640 supplemented with PS and 10% HS, counted and seeded at 30,000 cells/well in a 96-well round-bottom plate (VWR) to grow in 2D. The drug was added in three concentrations determined based on the IC50 and literature (Supplementary Table S3), as pretreatment of cells from PDOs when plated (day −3), or treated with the TILs (day 0). For 30,000 organoid cells, 100,000 immune cells were used. The drugs or vehicle DMSO were diluted in RPMI supplemented with PS and 10% HS.

On day −1, cells from PDOs were treated with 100 IU/mL IFN-γ (PeproTech, Cranbury, NJ, USA) for 24 h, as previously described elsewhere [21]. If the drug was added as pretreatment, it was added again to the same final concentrations in the IFN-γ treated conditions.

On day 0, the plate was centrifuged at 300× *g*, 5 min, 4 °C. Supernatant was discarded and tumor cells dissociated by adding 10 μL pre-warmed TrypLE (ThermoFisher Scientific) and incubated for 5 min at 37 °C, 5% CO_2_. Subsequently, 40 μL of the basis medium supplemented with 10% HS was added to each well, and the cells were gently resuspended. Drugs were prepared according to assay setup (Supplementary Table S3), and 100,000 TILs were directly added per well containing 30,000 PDO cells. TILs cultured alone or stimulated with phorbol myristate acetate (PMA; Sigma Aldrich, St. Louis, MO, USA) and Ionomycin (Sigma Aldrich) were used as negative and positive controls, respectively. To each well, 50 µL of Golgi solution was added consisting of 49.3 μL RPMI 1640 with 1% PS and 10% HS, 0.2 µL GolgiStop (BD Biosciences, San Jose, CA, USA), 0.2 µL GolgiPlug (BD Biosciences), and 0.3 µL anti-CD107a antibody (BD Biosciences), and the plate was incubated for 8 h at 37 °C, 5% CO_2_. For all conditions, the final volume was 200 µL/well.

After incubation, cells were washed twice with wash buffer. Then, 20 μL of Live/Dead solution (1:80 NIR/PBS) and 30 μL of an antibody mixture containing anti-CD4 (BD, 562970), CD8 (BD, Q10009), CD56 (BD, 563041), and CD3 (BD, 562280) antibodies were added to each well. The plate was incubated for 20 min at 4 °C, protected from light. Cells were washed and fixed using the FoxP3/Transcription Factor Staining Buffer Set (eBiosciences, Thermo Fisher Scientific). Plates were incubated for at least 40 min at room temperature in the dark or overnight at 4 °C. Subsequently, cells were permeabilized using freshly prepared and filtered permeabilization buffer (Perm Buffer; ThermoFisher Scientific), then washed and stained with 20 μL intracellular antibody mix (anti-TNF-α, IFN-γ, and CD137 antibodies) and incubated at 4 °C overnight. Cells were washed with permeabilization buffer and resuspended in 100 μL of wash buffer, and the samples were run on a NovoCyte Quanteon Flow Cytometer (Agilent).

### 2.16. Three-Dimensional Co-Culture Drug Response Assay

For the 3D co-culture drug screening assay, the cancer organoids were harvested, counted, and plated as described in the drug screening for treatment recommendations. For each dome containing 10,000 organoid cells, 100,000 immune cells were added for the co-culture setup. The screening setup included PDO and BME controls, as well as immune cell control conditions with activated, rested, and inhibited immune cells, as well as a high concentration of immune cells (300,000 cells/well). Immune cells were activated as described above and inhibited using 2.5 µg/mL Hydrocortisone (Solu Cortef, obtained from Herlev Hospital Pharmacy) in media for co-culture screening.

If a drug screening for clinical recommendation was performed, drug selection was based on these results. If the patient was deceased, a drug was selected based on the genomic profile. The drug was added in three concentrations determined based on the IC50 and literature (Supplementary Table S3), as pretreatment of PDOs when plated (day −3), or treated with the TILs (day 0).

Viability was measured using PrestoBlue as described above, and measurements were performed on day 0 prior to addition of TILs and drugs, and on day 3. Wells were washed after PrestoBlue reading on day 0 as described above. On day 3, the TILs were collected for each condition for post assay analysis. Triplicate wells were pooled, and wells were washed with warm organoid media. Microscope pictures were taken on day 0 and day 3 (OLYMPUS CKK53, OLYMPUS cellSens Entry v1.17).

### 2.17. T-Cell Bispecific Antibody Testing

A T-cell bispecific antibody (Cibisatamab; MedChemExpress, Monmouth Junction, NJ, USA) was evaluated using the 3D co-culture screening platform with organoid cultures and rested REP TILs. The setup was adapted to include controls for human IgG (Human IgG1 kappa, isotype control; MedChemExpress) for non-specific antibody binding control (Supplementary Table S3). T-cell bispecific antibody and human IgG control were reconstituted in sterile PBS prior to serial dilution, and PBS was used as vehicle control.

Viability was measured using PrestoBlue as described above, and measurements were performed on day 0 prior to the addition of TILs and drugs, and on day 3. Wells were washed after PrestoBlue reading on day 0 as described above.

### 2.18. Viability Calculations and Statistical Analysis

The 3D co-culture screening results were calculated as percentage viability on day 3 relative to day 0 in the same well. All experiments were performed with technical triplicates within each condition and three biological repeats. Wells with remaining PrestoBlue reagent from day 0 were excluded from calculations. Calculations were performed using Python (v3.12). Viability plots were constructed using matplotlib (v3.6.3) and statannotations (v0.7.2). Statistics were calculated for each drug condition and the vehicle control using Mann-Whitney test, for each individual repeat and biological replicates combined. Statistically significant differences were annotated as * *p* < 0.05; ** *p* < 0.001; *** *p* < 0.0001. Calculations were performed for each screening and repeats together in the combined analysis.

### 2.19. Post 3D Co-Culture Assay Analysis

Immune cells from the co-culture screenings were analyzed by flow cytometry staining using a panel designed to characterize T cells activation and cytotoxicity (Panel 6, Supplementary Table S1), including markers for T cell linage and function CD3 (PE-CF594), CD4 (BV711), CD8 (Qdot 605), CD56 (BV510), CD137 (APC), CD107a (BV421), and the live/dead marker NIR (APC-Cy7). Briefly, wash buffer consisting of FACS-PBS supplemented with 0.005% NaN_3_ (obtained from Herlev Hospital pharmacy, Cat. No. 800979) supplemented with 0.005 g/mL BSA (Sigma-Aldrich), and 0.4% EDTA (Invitrogen), was added to all samples and centrifuged at 300× *g*, for 5 min at 4 °C. Samples were transferred to a 96-well V-bottom plate, centrifuged, and 30 µL of NIR/surface antibody mixture was added to each sample and incubated for 20 min, 4 °C, protected from light. Cells were washed twice by adding 200 µL of cold wash buffer and centrifugation. After washing, cells were resuspended in 100 µL of wash buffer and samples acquired on the NovoCyte Quanteon (Agilent) flow cytometry instrument.

### 2.20. Flow Cytometry Data Processing and Analysis

Flow cytometry data was analyzed using the NovoExpress software 1.5.0 (Agilent, Santa Clara, CA, USA) and FlowJo (v10.10.0). Reactivity was defined in the 2D immune cell reactivity assay as the percentage of live CD3+ cells positive for at least two markers among CD137, CD107a, TNFa and IFNg. In the post-assay analysis, reactivity was instead defined as the percentage of live CD3+ cells positive for at least one marker among CD137 and CD107a. For clustering analyses, channel intensities from CD4+ and CD8+ T cells were exported. This input was further processed for clustering analysis using the Spectre package [31] (v1.2.0). Clustering was performed both separately by TIL type (yTILs and REP TILs) and panels (Panels 1 and 2). A subsample of 10,000 events (CD3^+^ T cells) was selected per patient. Annotation of clusters was based on CCR7 and CD45RA marker expression to identify different T cell differentiation states (T Naïve, TCM, TEM and TEMRA), and HLA-DR, CD57, and PD-1 in Panel 1, and LAG-3, BTLA, TIGIT, TIM-3, or PD-1 in Panel 2 (Supplementary Table S1), to define their activation status.

## 3. Results

### 3.1. Precision Cancer Medicine Immune Oncology: The PreCanIO Cohort

The PreCanIO cohort consists of 66 patients with various metastatic cancer types and biopsy sites (Figure 1B, Table 1). From the processed biopsies, we were able to obtain successful organoid cultures from 20 patients (30%), 19 patients (29%) were successful for yTIL cultures and 51 patients (77%) for immune cell cultures in total (yTIL and/or REP TIL), among which we obtained patient-matched organoid and immune cultures from 19 patients (29%). A detailed overview of the clinical and technical aspects of PreCanIO patients with successful organoid and immune cell cultures is provided in Table 1 and Table 2.

### 3.2. Tumor, Stromal, and Immune Cell Characterization of Organoid Cultures

The tumor microenvironment (TME) of cultured PDOs was characterized by flow cytometry to determine the relative proportions of cancer, immune, and stromal cells (Panel 3 and 4; Supplementary Table S1). Results suggested a lack of leukocyte populations in these PDOs, as absent or only low levels of CD45^+^ cells were detected (Supplementary Figure S1). Tumor cells were examined based on the expression of five tumor-associated markers (EpCAM, CEACAM5, epidermal growth factor receptor (EGFR), Mucin-1 (MUC1), and Claudin-6). Results showed that PDOs exhibited higher expression of CEACAM5 compared with control PBMCs, suggesting the presence of tumor cells with higher CEACAM5 expression, whereas the other four markers did not show higher expression in PDOs (Supplementary Figure S1). Within the CD45- non-immune cell population, cells with high expression of CEACAM5 and CD90 were determined to be tumor cells, whereas the fibroblast component was suggested based on high CD90 expression only. These findings indicate that immune cells do not persist within the cultured PDOs. Moreover, higher expression of CEACAM5 compared with control (PBMCs) was detected in the PDOs, suggesting potential relevance for therapeutic targeting.

### 3.3. Characterization of TILs

To obtain insight into the phenotypic composition of TILs from the PreCanIO cohort, T cell activation, differentiation and exhaustion (Panels 1 and 2; Supplementary Table S1) were investigated in patients with available yTILs (*n* = 14) and generated REP TILs (*n* = 10). Clustering analysis showed clear separation of CD4+ and CD8+ T cell clusters, indicating distinct phenotype profiles for the two T cell populations, for both yTILs (Figure 1C) and REP TILs (Figure 1D). Across the patient samples analyzed, CD4+ T cells formed larger clusters than CD8+ T cells within both yTIL and REP TIL populations (Figure 1C,D).

Cell clusters showed heterogeneity within the CD4+ and CD8+ T cell compartment, comprising distinct differentiation states including naive T cells (Naive), Central Memory T cells (CM), Effector Memory T cells (EM), and Terminally Differentiated Effector Memory T cells (EMRA) (Figure 1C,D). Additionally, a subset of these T cells was positive for activation markers (HLA-DR^+^, PD-1^+^, CD57+), indicating a successfully activated T cell population after the expansion (Panel 1). Furthermore, distinct subpopulations within both yTIL and REP TIL populations contained T cells expressing one or more exhaustion markers (BTLA, TIM-3, TIGIT, LAG-3, PD-1; Panel 2), suggesting the presence of phenotypically exhausted T cell subsets across both TIL types. Overall, this phenotypic analysis showed high heterogeneity of TILs across the tested patients.

A consistent pattern of higher rates of CD4+ expansion was observed within the CD3+ population of both in the yTILs and REP TILs (Figure 1C,D), as similarly observed in low-yield expansions [32,33].

### 3.4. Drug Screenings for Suggesting Therapy

**Figure 2:**
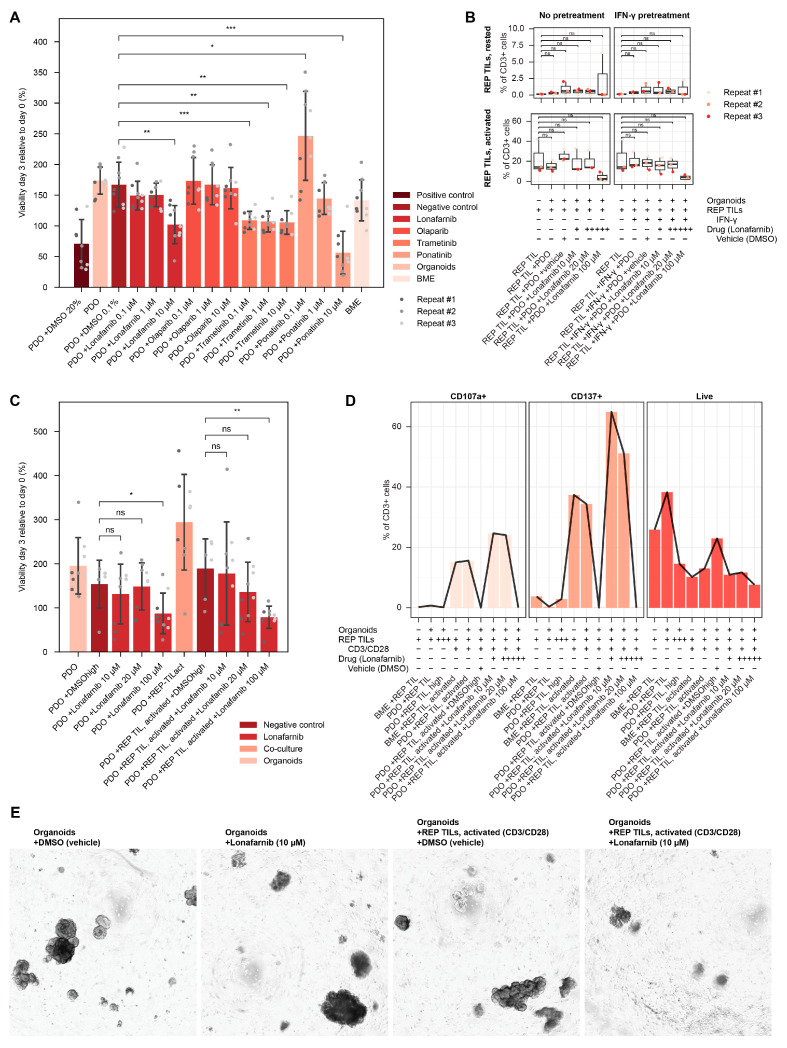
Drug screening results for patient A. Drug screening with Lonafarnib and three panel drugs (Olaparib, Trametinib, Ponatinib; 10 µM, 1 µM, 0.1 µM concentrations for all drugs) suggests response to Lonafarnib and Ponatinib (**A**). Lonafarnib was tested in the 2D co-culture reactivity assay, showing sustained T cell activation, but no evidence of increases in tumor-specific T cell reactivity (**B**). Lonafarnib was tested in 3D co-culture, showing significantly reduced viability in response to treatment, both in monoculture and co-culture with activated TILs (**C**). Post assay analysis shows tumor-independent T cell activation and a decrease in T cell viability and reactivity upon treatment with 100 µM Lonafarnib and the DMSO control (**D**). Microscope pictures (4× magnification) taken on day 3 of 3D co-culture drug screening show impaired organoid structures, both when treated as monoculture (left panels) and in co-culture (right panels) (**E**). DMSO vehicle control showed similar organoid presentation as PDO control condition. Images confirm TIL penetration of the BME domes. BME: basement membrane extract. DMSO: dimethyl sulfoxide. DMSOhigh: high concentration vehicle DMSO, used for highest drug concentration. ns: not significant. PDO: patient-derived organoid. REP: rapid expansion protocol. REP: rapid expansion protocol. TIL: tumor-infiltrating lymphocyte. *: *p* < 0.05. **: *p* < 0.001. ***: *p* < 0.0001. ns: not significant.

If the patient was still alive when organoids were growing, we performed drug screenings on organoids to suggest treatment for the patient. For the patient A (colorectal cancer), the genomic profile showed a high frequent NRAS p.Q61H mutation, and Lonafarnib was tested, as well as two kinase inhibitors and one PARP inhibitor selected from the panel drugs, in which two were selected based on the genomic profile (Olaparib based on two somatic pathogenic ATM mutations; Trametinib inhibiting MEK down stream of NRAS; Table 3). The combined screening results suggested a response to 10 µM Lonafarnib and 10 µM Ponatinib, with dose response, and a similar reduction in viability for all three concentrations of Trametinib, whereas we found no response to Olaparib (Figure 2A).

### 3.5. Co-Culture Drug Assays Show a Microenvironmental Impact on Drug Response

Next, we sought to investigate if our platform could be used to study the immunological changes upon ex vivo treatment of the isolated PDOs. Drug screenings were performed in 2D and 3D co-cultures with activated REP TILs. For patient A, mono-culture drug testing results (Figure 2A) served as the basis for further drug selection in co-culture treatment. Lonafarnib was tested in co-culture setups as it showed a consistent response when tested in monoculture, with a dose response pattern.

The 2D co-culture reactivity assay showed sustained T cell activation (Figure 2B) with no increase in reactivity observed when T cells were co-cultured with the PDOs. No changes were observed with the Lonafarnib treatment, but a decrease in T cell reactivity and viability was observed with the highest treatment concentration (40% average decrease in viability; *p* < 0.05).

In the 3D co-culture killing assay, Lonafarnib treatment resulted in a significant reduction in viability both in the presence and absence of activated TILs (Figure 2C). Additionally, we observe a disruption of organoid structures indicating cell death (Figure 2D).

Analysis of the TILs after the 3D assay showed preserved T cell activation, detectable after 72 h, with a marked decrease in T cell viability in the condition with the highest Lonafarnib concentration (Figure 2E). No increase in T cell reactivity was observed in the co-cultured conditions.

### 3.6. 3D Co-Culture Drug Screening Platform Applicability

To investigate the application of our drug testing platform, we investigated drug response in the 3D co-culture killing assays with two additional patients (patients B and C). Both patients were diagnosed with colorectal cancer (Table 2) and had different suggested treatments based on their genomic profile.

**Figure 3:**
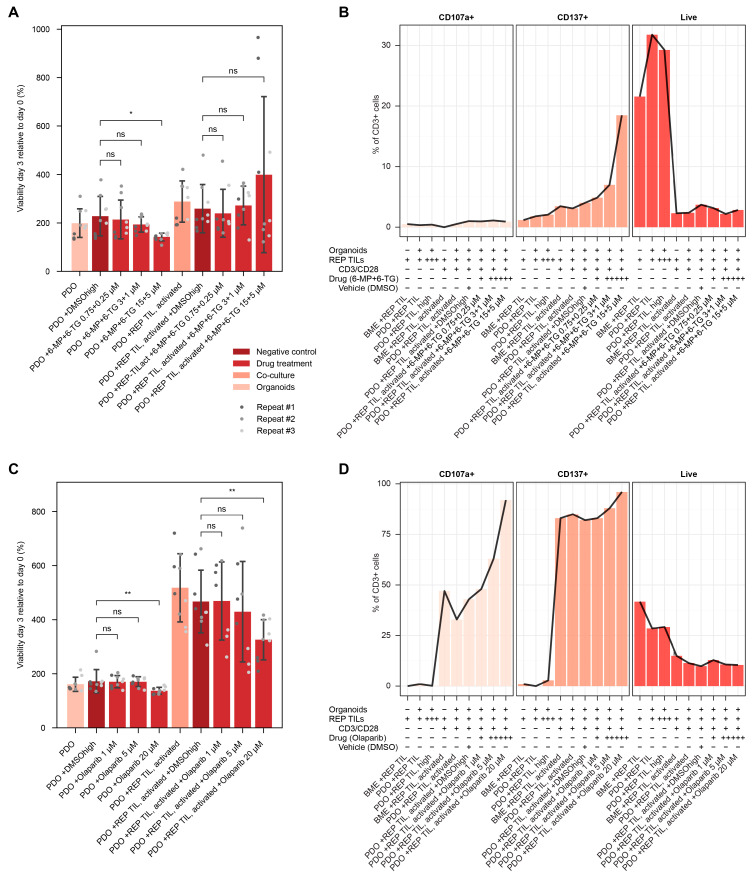
Immune cell effect on cancer drug response. 3D co-culture drug screening results for pretreatment of patient B (**A**) and co-culture treatment of patient C (**B**). Both testing strategies (pre-treatment of PDOs/treatment with TILs) and patients showed effects of TILs on drug response. Post 3D assay analysis showed a mild increase in post-assay reactivity for patient B (**C**) and patient C (**D**) in a dose-dependent manner. Statistical test: independent samples *t*-test, significance level *p* < 0.05. *: *p* < 0.05. **: *p* < 0.001. 6-MP: 6-mercaptopurine. 6-TG: 6-thioguanine. BME: basement membrane extract. DMSO: dimethyl sulfoxide. DMSOhigh: high concentration vehicle DMSO, used for highest drug concentration. ns: not significant. PDO: patient-derived organoid. REP: rapid expansion protocol. TIL: tumor-infiltrating lymphocyte. REP TIL, high: high concentration TIL condition (300,000 cells/well).

According to the genomic profile, patient B had an intermediate tumor-mutational burden (5–10 mutations/Mb) (Table 3), indicating eligibility for enrollment in the clinical trial NCT05276284 where patients are treated with a combination of 6-mercaptopurine (6-MP), 6-thioguanine (6-TG) and the PD-L1 inhibitor atezolizumab. We performed the assays by pretreatment with the drugs for 3 days prior to adding the activated TILs to the PDOs, as 6-MP should increase tumor neo-antigens [34]. Here, we found a significant reduction in tumor cell viability when treated in monoculture only (Figure 3A). Nonetheless, post-assay analysis showed a drug-dependent increase in the expression of T cell activation markers, but with an associated marked decrease in their viability (Figure 3B).

The genomic profile of patient C revealed homologous recombination deficiency (HRD) suggesting treatment with PARP inhibitors (Table 3), and Olaparib was investigated in our platform setup. We found significantly reduced viability in PDOs when treated with Olaparib compared with vehicle control, in both monoculture and co-culture treatment, in response to the highest tested concentration (Figure 3C). Interestingly, there was a greater reduction in viability in the co-culture condition, showing an impact of TILs presence on drug response and the potential synergy between PARPi and immune checkpoint inhibition [35]. Post-assay analysis showed again a dose-dependent increase in the expression of T cell activation markers, coupled with a decrease in viability (Figure 3D), consistent with the observations for patient B. These results are suggestive of a positive effect of personalized drug treatment over tumor-T cell interactions. The synergistic effect could be explained by the induction of drug-induced immunogenic tumor cell death, as observed with PARP inhibitors or tyrosine-kinase inhibitors [12,13].

### 3.7. Immune Cell Viability and Dome Penetration

As our setup includes distinct numbers of cells on day 0 and day 3 when viability is measured, our platform includes control immune conditions, for both technical and biological variability between patients. We found a higher viability for PDOs with high concentrations of normal rested immune cells (10,000 PDOs to 300,000 rested REP TILs) compared with the lower concentration used in the drug assay conditions (10,000 PDOs to 100,000 rested REP TILs). Activation states were investigated and showed lower viability in inhibited immune cells, compared with normal rested, and compared with activated immune cells (10,000 PDOs to 100,000 immune cells for all conditions). We found a consistent pattern across all three patients investigated in this study (Supplementary Figure S2). Taking immune cell proliferation from both microscope investigations and viability assay into account, this shows that immune cell activation impacts proliferation as well as the ability to penetrate the BME domes.

Dome penetration was particularly apparent for patient C (Supplementary Figure S2), possibly connected to the anti-tumor reactivity of the immune cells. We found that the immune cells penetrated the BME domes to a greater extent when activated compared with the rested condition in co-culture with the PDOs (Supplementary Figure S2). This confirms that the TILs are able to penetrate the BME used in our platform, and the extent may be dependent on anti-tumor reactivity and attraction, not physical hindrance.

### 3.8. T-Cell Bispecific Antibodies Hold Potential for Patients with Non-Reactive Immune Cells

One of the main limitations of current precision cancer medicine (PCM) pipelines is that therapy testing excludes the possibility of immune agents. Our platform showed potential effects of immune cells on drug response, and the co-culture setup could be applied for testing of immunotherapies as well as chemotherapies. The 3D setup is of particular relevance for the possibility to test T-cell bispecific antibodies, which could determine tumor-immune cell attraction in addition to tumor cell viability.

**Figure 4:**
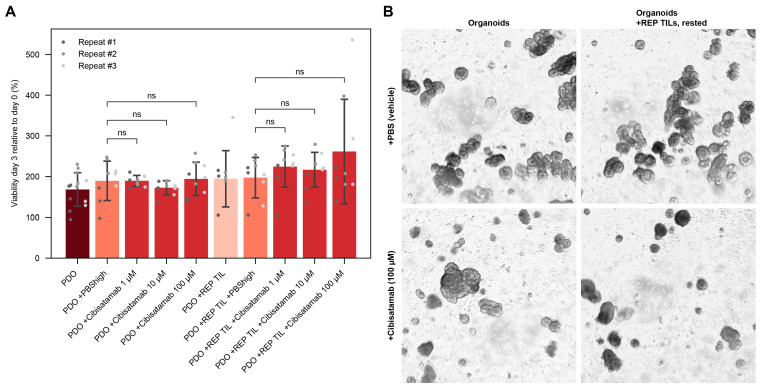
Testing T-cell bispecific antibody in 3D co-culture drug testing with patient A. T-cell bispecific antibody Cibisatamab was tested in 3D co-culture screenings, showing no significant alterations in viability in response to Cibisatamab (100 µM, 10 µM, 1 µM) (**A**). Microscope pictures show that PDO structures are maintained in both monoculture and co-culture treatment (4× magnification) (**B**). ns: not significant. REP: rapid expansion protocol. TIL: tumor-infiltrating lymphocyte.

The genomic profile from patient A showed high expression of CEACAM5 on RNAseq suggesting potential response to CEACAM5 directed immunotherapy (Table 3). The patient had previously been treated with the T-cell bispecific antibody Cibisatamab, binding CEACAM5 on cancer cells and CD3 on immune cells (Figure 4A; Supplementary Table S3), and we wanted to investigate if the same response was observed in our 3D co-culture setup.

The assay was performed with rested REP TILs, and Cibisatamab treatment showed increased proliferation in line with T cell activation. Cibisatamab did not alter the tumor cell viability in mono-culture treatment (Figure 4A), and microscope inspection confirmed maintained organoid structures (Figure 4B). Intriguingly, co-culture treatment showed a pattern of increased viability measurements with higher concentrations of Cibisatamab, likely due to TIL activation and increased proliferation, as PDO structures were not visually impaired in response to co-culture treatment. This suggests that Cibisatamab can be used to increase TIL activation, which could then be combined with another treatment, such as anti-PD-L1. In fact, the patient did not respond to Cibisatamab alone but showed an excellent response of sustained tumor control when treatment was combined with atezolizumab (anti-PDL1 treatment).

Furthermore, we investigated tumor marker expression in PDOs to see if this could explain the response (Supplementary Figure S3). We found that only a small portion of the cells expressed CEACAM5. This would explain the response observed in our in vitro assay, and emphasizes the need for further validation, e.g., expression at protein level, and not rely exclusively on genomic information alone.

## 4. Discussion

Patient-derived organoids (PDOs) are considered to be superior to other in vitro cancer cell cultures, due to the 3D structures of the organoids that mimic aspects of tumor growth and drug response [36,37]. However, immune cells are a dynamic part of the TME composition, known to influence therapy response [14,15]. Notably, our organoid cell composition analyses show low proportions of immune cells present in the organoids, whereas the tumoral and stromal components were found to be more contained. This emphasizes the importance and relevance of our immune-organoid co-culture platform. Additionally, functional testing, and particularly co-culture setups, are generally uncommon in precision medicine. Nonetheless, this is essential for evaluation of therapies where the immune cell component of the TME is of crucial importance, such as T-cell bispecific antibodies investigated in this study.

Here, we biopsy metastatic tumors and offer precision cancer medicine testing to metastatic cancer patients to identify effective therapies, test co-culture with patient-matched immune cells, and offer inclusion in relevant phase 1 clinical trials, where possible. Metastatic tumors often do not respond to treatment as predicted by primary tumor analysis [38], also highlighted by the distinct TME context at the metastatic compared with the primary tumor site [39], as well as the subpopulation of tumor cells that are responsible for the metastatic outgrowth. Metastatic biopsy collection can lead to improved understanding and treatment options for metastatic cancers that are not directly indicated by the primary tumor.

Our platform includes the possibility to examine individual patients’ cancer organoid response to various treatment options, including chemotherapies and immune agents. Notably, Olaparib was suggested based on the genomic profile of patient A, however, our results do not show a response. This highlights the importance of drug testing and including other indicators in addition to the genomic profiling alone for treatment decisions. Overall, our study illustrates the potential impact of immune cells on drug response. This also enables the possibility to investigate response to immunoagents, which have a more contextual response dependent on the immune cells’ properties that are included and evaluated in our platform. On the technical side, we additionally demonstrate a difference in the ability of TILs to penetrate the BME domes following their activation, although not triggered by a tumor-specific T cell activation.

The observed modulation of drug response in co-culture conditions suggests immune-mediated mechanisms. Activated TILs may influence tumor viability through cytokine secretion (e.g., IFN-γ, TNF-α), induction of immunogenic cell death or modulation of DNA damage response pathways. For Patient A, Olaparib was suggested based on two pathogenic ATM mutations rather than confirmed HRD status (HRD was not available). The lack of efficacy may indicate that ATM alterations alone are insufficient to confer PARP inhibitor sensitivity, or that compensatory repair mechanisms and bypass activation contribute to resistance, consistent with emerging evidence of PARP inhibitor resistance [12,13].

As the use of T-cell bispecific antibodies is emerging in patient care [40], our platform can be used to predict response to treatment, including the attraction of immune cells to the tumor site, their activation state, and reactivity against the tumor cells. T cell activation states can affect the TIL penetration of the BME domes, as illustrated in the 3D co-culture screening for patient C. In this study, organoids and immune cells of patient A were tested with Cibisatamab, a bispecific T-cell engaging antibody targeting CEACAM5 and CD3 that the patient had previously received with response. However, our results imply T cell functionality, and another treatment or combination with immunotherapy could be suggested [41]. Importantly, these results mimicked the in-patient scenario at the time of biopsy, in which the patient had progressed on the CEACAM5-directed T-cell engaging therapy. Cibisatamab had then been combined with anti-PDL1 treatment, which was highly effective, likely due to the increased T cell activation we observed in response to Cibisatamab. These findings again highlight how our screening setup mimics the clinical situation. Furthermore, our setup suggests panRAS inhibition as a potential next line therapy. The patient is currently being evaluated for inclusion in a trial with a panRAS inhibitor.

T-cell bispecific antibodies have previously been tested in ex vivo and in vitro settings [42]. A great advantage of our setup is the patient-matched material aspect, with direct transferrable value to the patient. Our results point toward a potential setup for drug testing and suggesting therapy for patients, evaluation of the tumor immune microenvironment effects on drug response, as well as a platform for testing response to bispecific antibodies. Our platform also extends to test immunotherapies and combining T-cell bispecific antibodies and immunotherapies such as anti-PD-L1, which are combined in clinical use. A possible technical limitation of our 3D co-culture drug testing platform is varying rates of TIL proliferation between patients, as well as TIL viability and possibly proliferation skewing the viability readouts of the organoids. However, proliferation is consistent within each patient, and the viability measurements are accompanied with microscope images for assessment of organoid fitness and morphology. Furthermore, to limit the variability between patients in our methodological setup, the same batch of feeder cells was used in all experiments. A major clinical obstacle for the complete PreCanIO pipeline is the success rate of establishing organoid cultures as well as the patient’s status when the organoid cultures have grown. All patients included in the study were late-stage cancer patients with exhausted treatment opportunities and multiple lines of therapy prior to biopsy. As a result, most patients are deceased before the organoid cultures were established and growing sufficiently to enable drug testing.

Our platform is promising for patients with an earlier diagnosis than those included in the current study. The precision cancer medicine pipeline and platform for therapy testing developed in this study holds potential for testing of drug candidates, immunotherapies, and T-cell bispecific antibodies for patients that have less aggressive disease and longer expected survival at the time of biopsy. Consideration of earlier stage cancer patients could relieve the economic burden on society by improving efficacy and overall outcome of patients at a less advanced stage.

## 5. Conclusions

Accurate models for precise testing are needed, and we believe this study is a valuable contribution for patients and further development of precision cancer medicine pipelines that take the tumor context into consideration. Our platform is promising for testing of non-immune and immune agents, including T-cell bispecific antibodies, in metastatic as well as less advanced cancer patients with direct translational value to the patients.

## Figures and Tables

**Table 1:** PreCanIO clinical and technical cohort description.

	PreCanIO Cohort	Patient-Matched Cultures ^(a)^
	*n* = 66	*n* = 19
Sex		
Female	36 (55%)	8 (42%)
Male	30 (45%)	11 (58%)
Age ^(b)^		
Median	58	60
Range	25–74	25–74
No. of prior treatment regimens ^(c)^		
Median	3	3
Range	0–8	2–7
Survival status		
Alive	14 (24%)	1 (5%)
Deceased	52 (76%)	18 (95%)
Time from biopsy to death ^(d)^		
Median	161	121
Range	18–1328	25–712

^(a)^ Portion of the complete PreCanIO cohort with successful patient-matched organoid and immune cell cultures (yTILs and/or REP TILs). ^(b)^ Age at time of biopsy. ^(c)^ Number of prior lines of treatment before admission to Phase 1 Unit. ^(d)^ Days from biopsy to death, median and range of deceased patients.

**Table 2:** Clinical and technical description of PreCanIO patients in study.

Patient	Gender	Age ^(a)^	Cancer Type		Biopsy Site	No. of Prior Treatment ^(b)^	Previous Immunotherapy		Survival Status
Patient A	M	58	CRC	Colon	Lung	2	Yes	Cibisatamab + atezolizumab	Alive
Patient B	M	64	CRC	Colon	Liver	3	No	–	Deceased
Patient C	M	60	CRC	Rectal	Liver	6	No	–	Deceased

^(a)^ Age at time of biopsy. ^(b)^ Number of prior lines of treatment before admission to Phase 1 Unit. CRC: colorectal cancer.

**Table 3:** Genomic information on selected PreCanIO study patients.

Patient	Selected Somatic Mutations ^(a)^			TMB ^(b)^ (mut/Mb)	Selected CNVs ^(c)^	HRD Status	Selected Gene Exp. ^(d)^	Germline Mutations ^(e)^		
	Gene	Mutation Type	Classification					Gene	Mutation Type	Classification
Patient A	*NRAS*	Missense	Pathogenic	6.6	*NBN* amp. *MYC* amp.	NA	*CEACAM5* *MYC* *VEGFA*	None		
	*ATM*	Frameshift	Likely pathogenic							
	*ATM*	Nonsense	Likely pathogenic							
Patient B	*KRAS*	Missense	Pathogenic	7.0	None	Negative	*ERBB2* *ERCC1* *FLT1/4* *MET* *PTEN* *TP53*	*MUTYH* (het)	Missense	Pathogenic
	*TP53*	Missense	Pathogenic							
	*TGFBR1*	Missense	Likely pathogenic							
	*APC*	Frameshift	Likely pathogenic							
Patient C	*NRAS*	Missense	Pathogenic	6.8	*NBN* amp.	Positive	*AURKA* *CEACAM5* *ERBB2* *GNAS* *TP53*	*BRCA1* (het)	Nonsense	Pathogenic
	*APC*	Nonsense	Pathogenic							
	*TP53*	Missense	Likely pathogenic							

^(a)^ Selected somatic mutations from tumor-normal analysis of WGS data. ^(b)^ Tumor mutational burden was calculated as number of non-synonymous somatic mutations per megabase (mut/Mb). ^(c)^ Copy number variations were assessed from the genomic profiles and selected amplifications are shown. ^(d)^ Expression levels of selected genes were obtained from RNAseq data. A subset of highly expressed genes from the genomic reports are shown. ^(e)^ Germline variants were called from germline WGS data. amp: amplification, >5 copies. CNV: copy number variations. HRD: homologous recombination deficiency. NA: not available. TMB: tumor mutational burden, calculated as number of non-synonymous somatic mutations per megabase (mut/Mb), het: heterozogous.

## Data Availability

Source data is provided with this publication. Clinical and patient information can be made available upon reasonable request.

## Supplementary Materials

The following supporting information can be downloaded at: https://www.mdpi.com/article/10.3390/cells15030259/s1, Figure S1: Expression profiling of cancer, stromal, and immune cell markers in PDOs selected from PreCanIO patients; Figure S2: Immune cell conditions in 3D co-culture screenings; Figure S3: Expression profiling of cancer, stromal and immune cell markers in PDOs from patient A; Table S1: Flow cytometry antibody panels; Table S2: Panel of kinase inhibitors; Table S3: Specific drugs suggested based on genomic profile; File S1: Raw flow cytometry data.

## Abbreviations

The following abbreviations are used in this manuscript:

6-MP	6-mercaptopurine
6-TG	6-thioguanine
BME	Basement membrane extract
BSA	Bovine serum albumin
CNV	Copy number variation
CM	Central memory T cell
DMSO	Dimethyl sulfoxide
EGFR	Epidermal growth factor receptor
EM	Effector memory T cell
EMRA	Terminally differentiated effector memory T cell
HRD	Homologous recombination deficiency
HS	Human AB serum
PBMC	Peripheral blood mononuclear cell
PCM	Precision cancer medicine
PBS	Phosphate buffered saline
PDO	Patient-derived organoid
PMA	Phorbol myristate acetate
PS	Penicillin-Streptomycin
RM	Rapid expansion medium
REP	Rapid expansion protocol
TIL	Tumor-infiltrating lymphocyte
TMB	Tumor mutational burden
TME	Tumor microenvironment
yTIL	Young tumor-infiltrating lymphocyte
