# Supplementary material for: Immune Cell Modulation of Patient-Matched Organoid Drug Response in Precision Cancer Medicine Platform
Source: Cells. 2026 Jan 29;15(3):259. doi: 10.3390/cells15030259 (PMC12897217; doi:10.3390/cells15030259)
Supplement: Supplementary file 1 [file cells-15-00259-s001.zip › cells-4015292_suppl_text_revised.pdf]

cells

MDPI

Article

Immune cell modulation of patient-matched organoid drug response in precision cancer medicine platform

Silje Kjølle [1], Mario Presti [2], Jéssica de Pina Roque [1], Lina Hua Bisgaard [2], Darío Beceiro Ramos [1], Kamilla Westarp Zornhagen [1,3], Christina Westmose Yde [4], Ane Yde Schmidt [4], Perrine Verdys [2], Martin Højgaard [3], Ulrik Lassen [3], Inge Marie Svane [2], Kristoffer Staal Rohrberg [3#], Marco Donia [2#] and Janine T Erler [1#*].

Supplementary

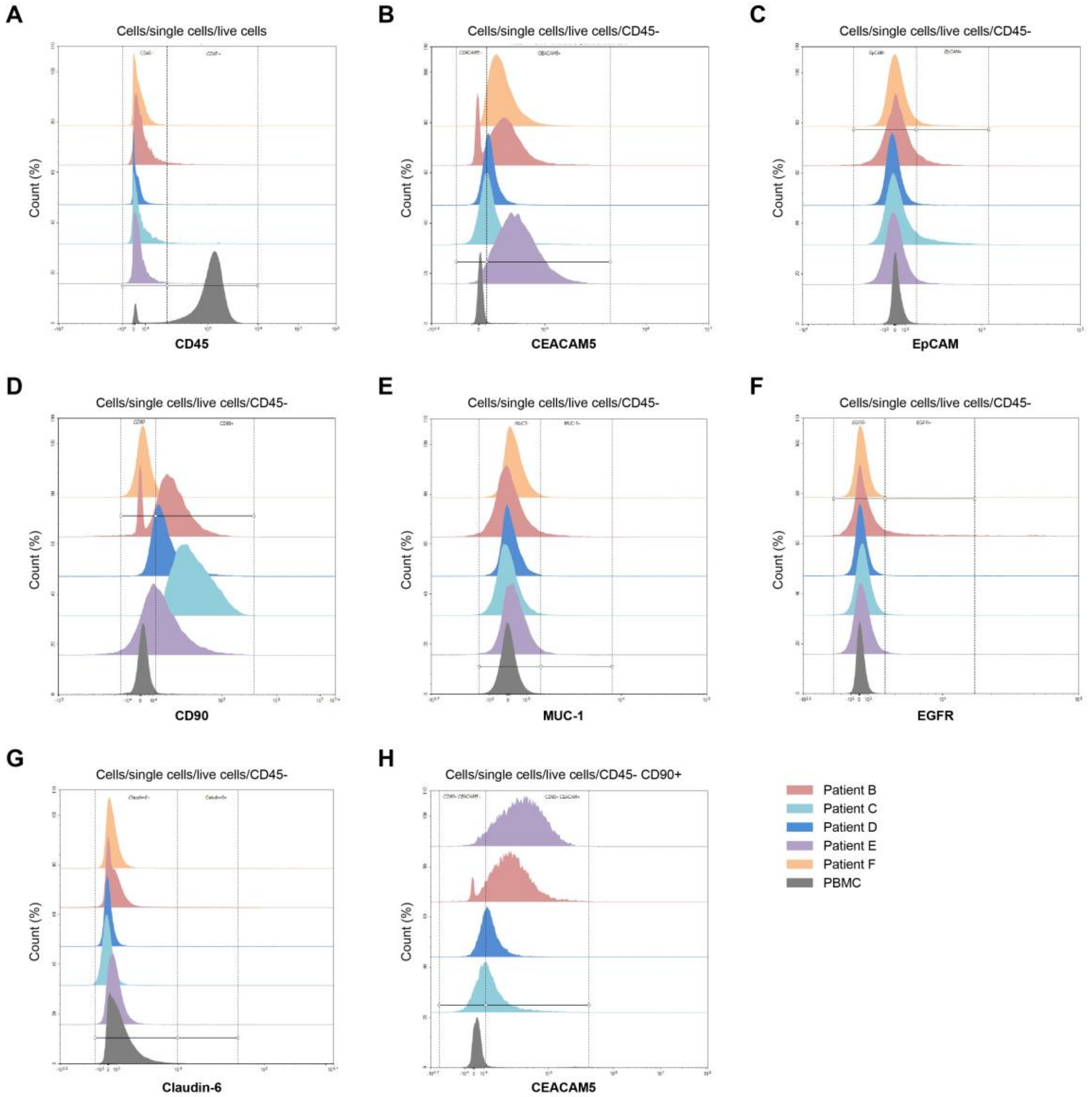

Supplementary Figure S1: Expression profiling of cancer, stromal, and immune cell markers in PDOs selected from PreCanIO patients. Flow cytometry histograms showing expression of CD45, CEACAM5, EpCAM, CD90, MUC-1, EGFR and Claudin-6 in PDOs from PreCanIO patients (n=5). Gating strategy is indicated above each plot, and vertical lines denote negative and positive thresholds. All histograms are normalized to mode. PBMCs served as positive control for CD45⁻ gating

and negative control for tumor-associated markers. PBMC: peripheral blood mononuclear cell. PDO: patient-derived organoid.

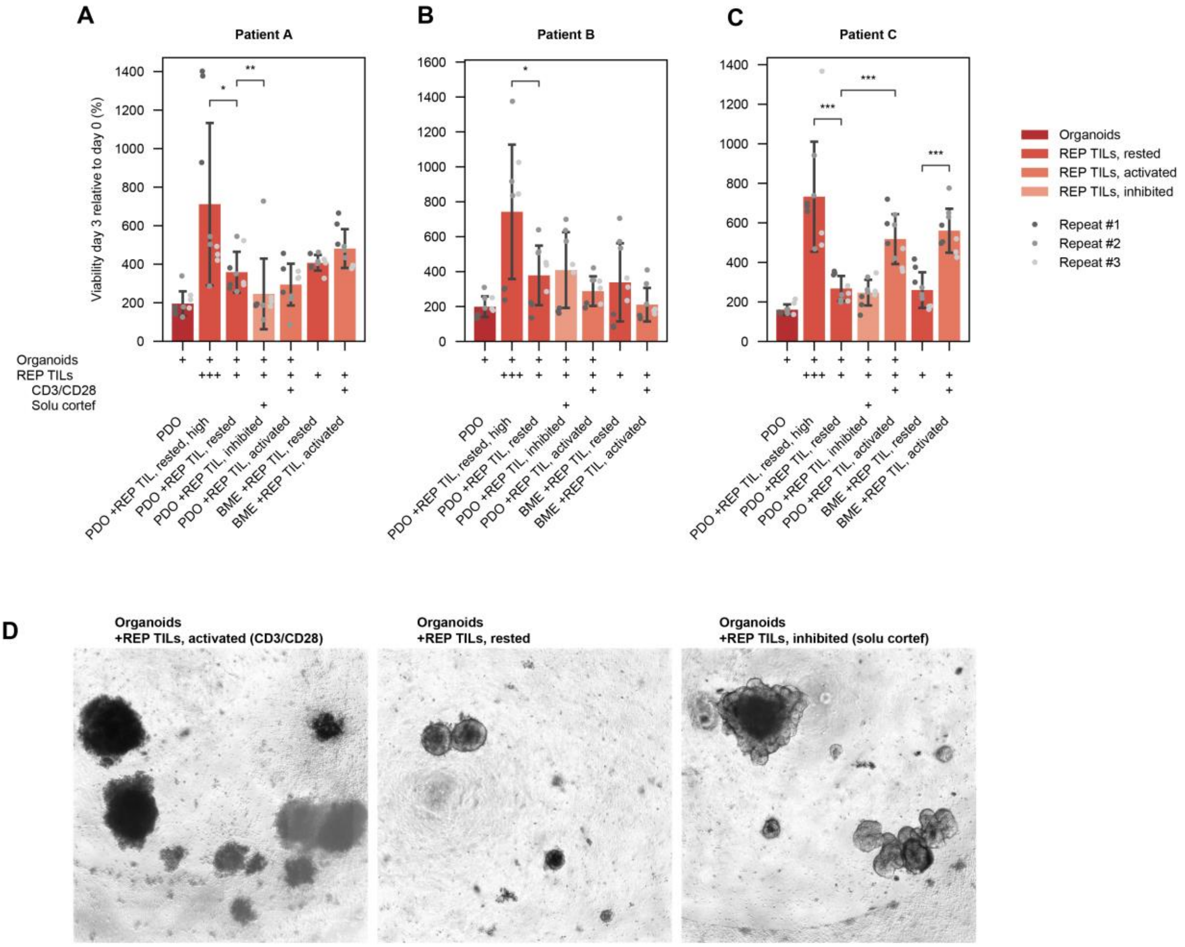

Supplementary Figure S2: Immune cell conditions in 3D co-culture screenings. Immune cell control conditions show higher viability measured for REPhigh (300,000 cells/well) than REPlow (100,000 cells/well) for rested immune cells, as well as higher viability for activated immune cells (CD3/CD28 activated, 100,000 cells/well), with consistent patterns for patient A (**a**), patient B (**b**), and patient C (**c**) (all 10,000 PDOs/well). Patient C illustrates differences in immune cells' ability to penetrate BME domes in 3D co-culture screening setups for activated (left), rested (middle) and inhibited (right) REP TILs in BME domes containing PDOs (**d**). BME: basement membrane extract. PDO: patient-derived organoid. REP: rapid expansion protocol. TIL: tumor infiltrating lymphocyte.

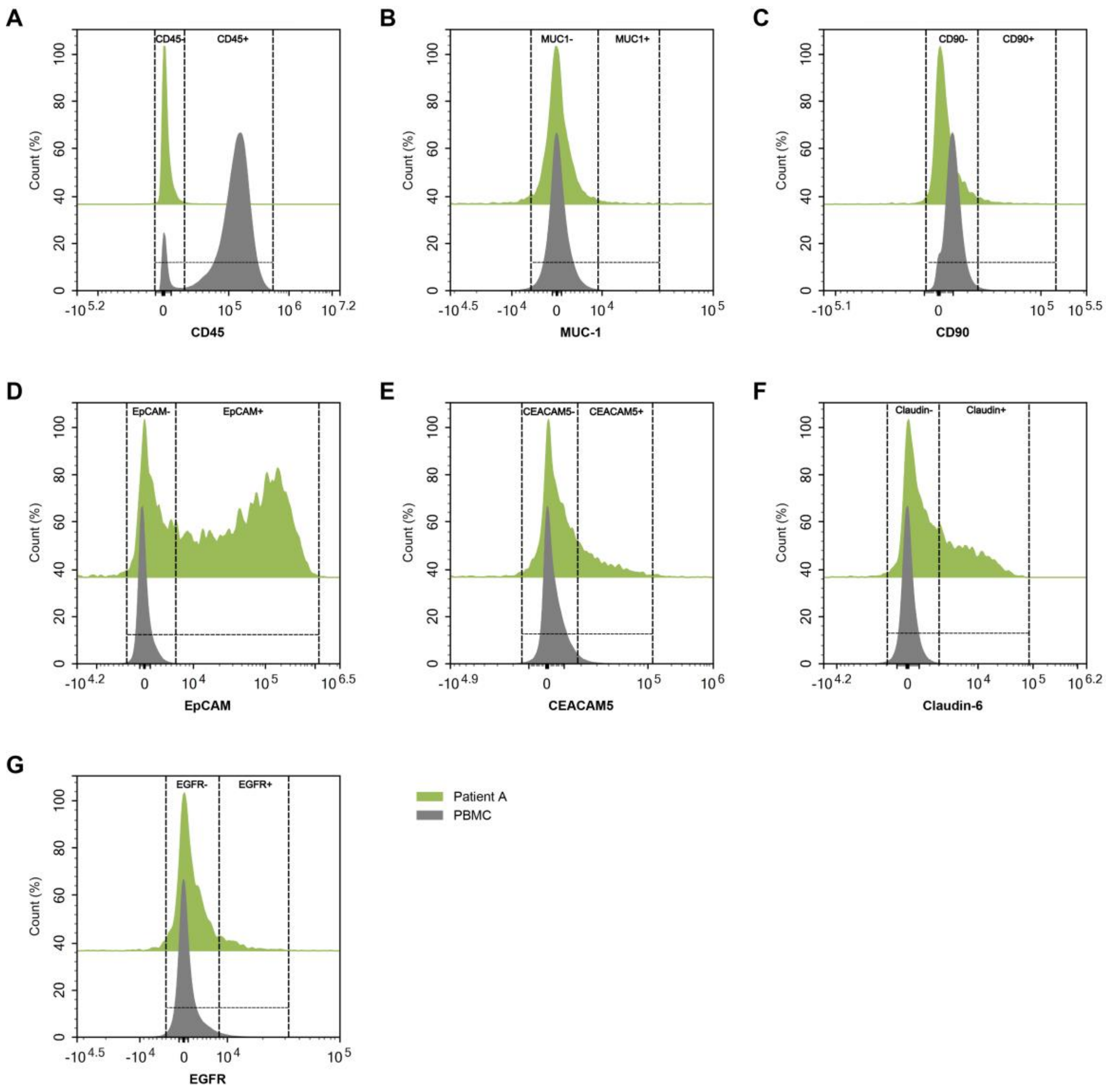

Supplementary Figure S3: Expression profiling of cancer, stromal, and immune cell markers in PDOs from patient A. Flow cytometry histograms showing expression of CD45, CEACAM5, EpCAM, CD90, MUC-1, EGFR and Claudin-6 in PDOs from PreCanIO patient A. Gating strategy is indicated above each plot, and vertical lines denote negative and positive thresholds. All histograms are normalized to mode. PBMCs served as positive control for CD45$^-$ gating and negative control for tumor-associated markers. PBMC: peripheral blood mononuclear cell. PDO: patient-derived organoid.

Supplementary Table S1: Flow cytometry antibody panels.

Target	Fluorophore	Clone	Distributor	Cat. No.	Dilution
Panel 1: Phenotyping TILs					
CD57	FITC	NK-1	BD	555619	1:100
HLA-DR	PerCP-Cy5.5	G46-6	BD	560652	1:25
CD28	APC	CD28.2	BD	559770	1:25
CD8	APC-R700	RPA-T8	BD Horizon	565165	1:100
Live/Dead-NIR	APC-Cy7		Invitrogen	L34976	1:1000
CD45RO	BV421	UCHL1	BD	562641	1:25
CD4	BV510	SK3	BD	562970	1:50
CD56	BV605	NCAM16.2	BD	562779	1:100
CD45RA	BV650	HI100	BD	563963	1:50
CD27	BV711	M-T271	BD	563167	1:100
CD3	BV786	Sk7	BD	345765	1:50
CCR7	PE	G043H7	BioLegend	353204	1:25
PD-1	PE-CF594/da	EH12.2H7	BioLegend	329940	1:50
CD95	PE-Cy7	DX2	BD	561633	1:25
Panel 2: Phenotyping TILs					
LAG3	FITC	11C3C65	BioLegend	369308	1:50
CD56	BB700	NCAM16.2	BD	566573	1:100
BTLA	APC	J168-540	BD	564800	1:50
CD8	APC-R700	RPA-T8	BD Horizon	565165	1:100
Live/Dead-NIR	APC-Cy7		Invitrogen	L34976	1:1000
TIGIT	BV421	741182	BD	747844	1:50
CD4	BV510	SK3	BD	562970	1:50
CD103	BV605	Ber-ACT8	BioLegend	350218	1:25
CD45RA	BV650	HI100	BD	563963	1:50
TIM3	BV711	7D3	BD	565566	1:50
CD3	BV786	Sk7	BD	345765	1:50
CCR7	PE	G043H7	BioLegend	353204	1:25
PD-1	PE-CF594/da	EH12.2H7	BioLegend	329940	1:50
CD29	PE-AF700	HI29a	AAT Bioquest	102901P1	1:25
CD39	PE-Cy7	eBioA1	Invitrogen	25-0399-42	1:50
Unstained Panel 1 (control)					
CD8	APC-R700	RPA-T8	BD Horizon	565165	1:100
Live/Dead-NIR	APC-Cy7		Invitrogen	L34976	1:42 / 1:1000
CD4	BV510	SK3	BD	562970	1:50
CD56	BV605	NCAM16.2	BD	562779	1:100
CD3	BV786	Sk7	BD	345765	1:50
Unstained Panel 2 (control)					
CD56	BB700	NCAM16.2	BD	566573	1:100
CD8	APC-R700	RPA-T8	BD Horizon	565165	1:100
Live/Dead-NIR	APC-Cy7		Invitrogen	L34976	1:1000
CD4	BV510	SK3	BD	562970	1:50
CD3	BV786	Sk7	BD	345765	1:50
Panel 3: Tumor cell characterization in PDOs					
CD45	FITC	2D1	BD	345808	1:50
MUC-1	RB780	HMFG2	BD	756292	1:50
Live/Dead-NIR	APC-Cy7		Invitrogen	L34976	1:1000

Claudin-6 (CLDN6)	Alexafluor 647	342927	RD	FAB3656R	1:20
CD66e (CEACAM5)	VioBright V423	REA876	Miltenyi Bio-tec	130130757	1:25
CD90	BV510	5E1O	BD	563070	1:25
EpCAM	PE	CO17-1A	BioLegend	369805	1:25
EGFR	PE-Cy7	AY13	BioLegend	352909	1:10
Panel 4: Immune cell characterization in PDOs					
CD45	FITC	2D1	BD	345808	1:50
HLA-DR	APC-R700	G46-6	BD	565127	1:25
Live/Dead-NIR	APC-Cy7		Invitrogen	L34976	1:1000
CD66b	VioBright V423	REA306	Miltenyi Bio-tec	130128268	1:25
CD16	BV510	3G8	BioLegend	302048	1:25
CD11c	BV650	B-ly6	BD	563404	1:12,5
CD19	BV711	SJ25C1	BD	563036	1:25
CD3	BV786	Sk7	BD	345765	1:50
CD14	PE-CF594	MoP9	BD	562335	1:125
CD163	Pe-Cy7	GHI/61	BioLegend	333613	1:100
Panel 5: TIL reactivity					
CD107a	BV421	H4A3	BD	562623	1:200
CD4	BV711	SK3	BD	563028	1:30
CD8	Qdot 605	3B5	Invitrogen	Q10009	1:150
CD56	BV510	NCAM16.2	BD	563041	1:20
CD3	PE-CF594	UCHT1	BD	562280	1:37,5
TNF-α	APC	MAb11	BioLegend	502912	1:20
IFN-γ	PE-Cy7	X	BD	557643	1:13.5
CD137 (4-1BB)	PE	X	BD	555956	1:20
NIR	APC-Cy7		Invitrogen	L34976	1:1000
Panel 6: Post reactivity staining					
CD3	PE-CF594	UCHT1	BD	562280	1:25
CD137	APC	4B4-1	BD	555956	1:13.5
Live/Dead-NIR	APC-Cy7		Invitrogen	L34976	1:1000
CD107a	BV421	H4A3	BD	562623	1:20
CD56	BV510	NCAM16.2	BD	563041	1:13.5
CD8	Qdot 605	3B5	Invitrogen	Q10009	1:100
CD4	BV711	SK3	BD	563028	1:20

Supplementary Table S2: Panel of kinase inhibitors.

Drug name	Mechanism	Target(s)	IC50[a, b]	*In vitro* concentration range[b]	Incubation time[b]
Bosutinib	Kinase inhibitor. Promotes autophagy.	Abl	1 nM	1 µM	72 hours
		Scr	2 nM		
Dasatinib	Multi-target kinase inhibitor. Induces autophagy and apoptosis.	Abl	<1 nM	32 nM	72 hours
		Src	0.8 nM		
		c-Kit	79 nM		
Fosfamatinib	Prodrug for R406. Induce apoptosis.	SYK	41 nM	NA	NA
Lenvatinib	Kinase inhibitor. Anti-angiogenesis.	VEGFR2	4 nM	0-10 µM	72 hours
		VEGFR3	5.2 nM		
		VEGFR1	22 nM		
		PDGFRB	39 nM		
		FGFR1	46 nM		
		PDGFRA	51 nM		
		Kit	100 nM		
Olaparib	Kinase inhibitor, selective. Induces autophagy associated with mitophagy.	PARP1	5 nM	1-300 nM	7-14 days
		PARP2	1 nM		
Pazopanib	Multi-target kinase inhibitor. Induces autophagy.	VEGFR1	10 nM	NA	NA
		VEGFR2	30 nM		
		VEGFR3	47 nM		
		PDGFR	84 nM		
		FGFR	74 nM		
		c-Kit	140 nM		
		c-Fms/CSF1R	146 nM		
Ponatinib	Multi-target kinase inhibitor. Inhibits autophagy.	Abl	0.37 nM	0-625 nM	72 hours
		PDGFRα	1.1 nM		
		VEGFR2	1.5 nM		
		FGFR1	2.2 nM		
		Src	5.4 nM		
Trametinib	Kinase inhibitor, selective. Activates autophagy, induces apoptosis.	MEK1	0.92 nM	10 µM	3-4 days
		MEK2	1.8 nM		

(a) IC50 determined in cell-free assay.

(b) IC50, concentration ranges and incubation times retrieved from Selleckchem.com where available.

Supplementary Table S3: Specific drugs suggested based on genomic profile.

Patient	Drug name	Mechanism	Target(s)	IC50[(a)]	Assay conc.[(b,c)]	Treatment
Patient A	Lonafarnib	panRAS inhibitor. Antiproliferative, induces apoptosis.	H-ras	1.9 nM	100 μM<br>20 μM<br>10 μM	Treatment with immune cells (day 0)
			K-ras-4B	5.2 nM		
			N-ras	2.8 nM		
	Cibisatamab	Bispecific T cell engager	CEACAM5	–	100 μM<br>10 μM<br>1 μM	Treatment with immune cells (day 0)
			CD3	–		
Patient B	6-Mercaptopurine[(d)]	DNA/RNA synthesis inhibitor, immune-suppressive.	PRPP amido-transferase		15 μM<br>3 μM<br>0.75 μM	Pretreatment of organoids (day -3)
	6-Thioguanine[(d)]	DNA methyl-transferase inhibitor.	DNMT1		5 μM<br>1 μM<br>0.25 μM	
Patient C	Olaparib	PARP inhibitor. Induces autophagy and associated mitophagy.	PARP1	5 nM	20 μM<br>5 μM<br>1 μM	Treatment with immune cells (day 0)
			PARP2	1 nM		

(a) IC50 determined in cell-free assay.

(b) Assay concentrations determined based on literature and IC50 in cell-free assays.

(c) Assay concentration of Cibisatamab is based on literature.

(d) 6-Mercaptopurine and 6-Thioguanine were dosed in a 3:1 ratio based on clinical dosing.
